# High detectability with low impact: Optimizing large PIT tracking systems for cave‐dwelling bats

**DOI:** 10.1002/ece3.5482

**Published:** 2019-09-15

**Authors:** Emmi van Harten, Terry Reardon, Lindy F. Lumsden, Noel Meyers, Thomas A. A. Prowse, John Weyland, Ruth Lawrence

**Affiliations:** ^1^ Department of Ecology, Environment and Evolution La Trobe University Bundoora Vic. Australia; ^2^ South Australian Museum Adelaide SA Australia; ^3^ Department of Environment, Land, Water and Planning Arthur Rylah Institute for Environmental Research Heidelberg Vic. Australia; ^4^ La Trobe University Bendigo Vic. Australia; ^5^ School of Mathematical Sciences The University of Adelaide Adelaide SA Australia; ^6^ School of Natural and Built Environments University of South Australia Mawson Lakes SA Australia

**Keywords:** bats, mark‐recapture, microchip, PIT‐tag, radio‐frequency identification, wildlife tracking

## Abstract

Passive integrated transponder (PIT) tag technology permits the “resighting” of animals tagged for ecological research without the need for physical re‐trapping. Whilst this is effective if animals pass within centimeters of tag readers, short‐distance detection capabilities have prevented the use of this technology with many species. To address this problem, we optimized a large (15 m long) flexible antenna system to provide a *c*. 8 m^2^ vertical detection plane for detecting animals in flight. We installed antennas at two roosting caves, including the primary maternity cave, of the critically endangered southern bent‐winged bat (*Miniopterus orianae bassanii*) in south‐eastern Australia. Testing of these systems indicated PIT‐tags could be detected up to 105 cm either side of the antenna plane. Over the course of a three‐year study, we subcutaneously PIT‐tagged 2,966 bats and logged over 1.4 million unique detections, with 97% of tagged bats detected at least once. The probability of encountering a tagged bat decreased with increasing environmental “noise” (unwanted signal) perceived by the system. During the study, we mitigated initial high noise levels by earthing both systems, which contributed to an increase in daily detection probability (based on the proportion of individuals known to be alive that were detected each day) from <0.2 (noise level ≥30%) to 0.7–0.8 (noise level 5%–15%). Conditional on a low (5%) noise level, model‐based estimates of daily encounter probability were highest (>0.8) during peak breeding season when both female and male southern bent‐winged bats congregate at the maternity cave. In this paper, we detail the methods employed and make methodological recommendations for future wildlife research using large antennas, including earthing systems as standard protocol and quantifying noise metrics as a covariate influencing the probability of detection in subsequent analyses. Our results demonstrate that large PIT antennas can be used successfully to detect small volant species, extending the scope of PIT technology and enabling a much broader range of wildlife species to be studied using this approach.

## Graphical abstract

Passive integrated transponder tag (PIT) technology permits the “resighting” of animals tagged for ecological research without the need for physical re‐trapping; however, short‐distance detection capabilities of antennas have prevented the use of this technology with many species. In a three‐year study, we optimized a large 15 m loop antenna to detect animals in flight, subcutaneously PIT‐tagged 2,996 cave‐dwelling bats, and logged over 1.4 million detections, with 97% of the tagged individuals being detected at least once. Results demonstrate that large PIT antennas can successfully be used to detect small volant species, extending the scope of PIT technology and enabling a much broader range of wildlife species to be studied using this approach.

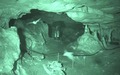

## 1 INTRODUCTION

Ecological research often requires wild‐living individuals to be marked and then recaptured, tracked, or re‐sighted over time. Applying these techniques can be challenging due to low recapture rates, technological constraints, welfare considerations, and the need to minimize disturbance to threatened populations (Cooke et al., 2013; Schorr, Ellison, & Lukacs, 2014). A wide range of marking and tagging techniques are available to monitor wildlife, including mutilation (e.g., toe clipping or ear notching), banding, radio‐transmitters, acoustic tags, and bio‐loggers (Bino, Kingsford, Grant, Taylor, & Vogelnest, 2018; Murray & Fuller, 2000; O'Mara, Wikelski, & Dechmann, 2014; Perry, Wallace, Perry, Curzer, & Muhlberger, 2011; Walker, Trites, Haulena, & Weary, 2012; Wilmers et al., 2015). Marking can, however, have potential negative effects on wildlife, including injury, reduced survival and reproduction rates, and changes to behavior and movement (Baker et al., 2001; Bodey et al., 2018; Griesser et al., 2012; Murray & Fuller, 2000; Rosen, Gerlinsky, & Trites, 2018). Many methods also necessitate repeated trapping and handling, which is labor intensive and, despite continued effort being made to minimize impacts on wildlife, each trapping typically induces stress in trapped individuals (Gelling, McLaren, Mathews, Mian, & Macdonald, 2009; Lynn & Porter, 2008; Reeder, Kosteczko, Kunz, & Widmaier, 2004) and has inherent (but low) mortality rates (Blomberg, Davis, Mangelinckx, & Sullivan, 2018; Lemckert, Brassil, Kavanagh, & Law, 2006).

Exemplifying these challenges is the study of bats, of which a large proportion are small insectivorous species, that are cryptic, highly mobile, and difficult to recapture (Schorr et al., 2014). These ecological and behavioral traits pose practical challenges and ethical considerations for marking and tracking individuals. Almost 40% of all assessed bat species worldwide are considered threatened, near threatened, or data deficient under IUCN criteria (IUCN, 2018). Therefore, it is critical to improve these techniques to enable effective research approaches without significantly impacting the bats' viability.

Banding has been used to mark bats since 1910s (Allen, 1921), but can cause significant injury and lower survival in some species (Baker et al., 2001). Another alternative is radio‐tracking; however, a comprehensive review has found that most radio‐tracking devices used to study bats are too heavy, are being used with minimal ethical justification, and remain attached for an average of just 9 days (O'Mara et al., 2014). A more recent innovation for the use on small bats has been miniaturized GPS tags; however, currently this has only been successfully attempted with the use of anesthesia and sutures to attach the loggers—and battery life, tag weight and recapture rates remain ongoing issues (Castle, Weller, Cryan, Hein, & Schirmacher, 2015; Weller et al., 2016).

An alternative to these marking and tracking methods are passive integrated transponder (PIT) tags, which weigh as little as 0.1 g, which is well under the 5% of body mass “rule” recommended for bats under 70 g (Aldridge & Brigham, 1988; Neubaum, Neubaum, Ellison, & O'Shea, 2005). To date, PIT‐tags have shown no apparent effect on body condition or reproductive success of small bats (Rigby, Aegerter, Brash, & Altringham, 2012).

PIT‐tags are glass‐encapsulated microchips that are injected into an animal and lay dormant until they are activated by a hand scanner or antenna system, which reads the tag's globally unique identification number using radio‐frequency identification (RFID). By positioning antenna systems at key locations, individuals can be passively tracked for a lifetime with just a single trapping event. PIT‐tag technology has been used extensively in fish research since 1980s and has also been used to study birds, reptiles, amphibians, invertebrates, and mammals (Gibbons & Andrews, 2004; Schlicht & Kempenaers, 2018; Soanes, Vesk, & Ree, 2015; Unger, Burgmeier, & Williams, 2012). PIT‐tag technology has advanced the study of movement patterns and survival of wildlife; however, a major limitation of this technology has been low detection distance, with tagged individuals normally needing to pass within 30 cm or less of an antenna to be detected (Adams & Ammerman, 2015; Gibbons & Andrews, 2004; Norquay & Willis, 2014).

To date, microbat studies using PIT‐tags and passive detection have been limited to close‐range applications, typically using loop antennas at small roost entrances, such as tree hollows (Garroway & Broders, 2007; O'Donnell, Edmonds, & Hoare, 2011; Toth, Dennis, Pattemore, & Parsons, 2015), bat boxes (Godinho, Lumsden, Coulson, & Griffiths, 2015; Kerth & König, 1996; Kerth & Reckardt, 2003), or small building entrances (Ellison, O'Shea, Neubaum, & Bowen, 2007; O'Shea et al., 2010; Safi, König, & Kerth, 2007). Bats have also successfully been detected at an artificial water source when tagged individuals came within 15 cm of a submerged plate antenna (Adams & Hayes, 2008). Large roost entrances, such as with caves, provide additional challenges for using PIT antennas due to the detection ranges required. PIT antennas have been installed on timber frames partly covered with mesh to modify the cave exit and funnel the bats through small “windows” (Britzke, Gumbert, & Hohmann, 2014), or by using a serpentine antenna configuration that zig‐zags across the cave entrance (Adams & Ammerman, 2015). A drawback of these approaches is that they altered the flight path of the bats and led to short‐term responses and effects such as circling, landing on the infrastructure, avoidance behavior, and wing‐strikes. Furthermore, some bats have limited tolerance to structural changes at their roost entrances. For example, gates at caves and mines have caused some bats to modify their behavior and have been linked to declines in numbers and, in some cases, total site abandonment (Pugh & Altringham, 2005; Slade & Law, 2008; Tuttle, 1979). Increased predation risk can also result with predators using infrastructure to catch bats exiting the roost (White & Seginak, 1987).

The critically endangered southern bent‐winged bat (*Miniopterus orianae bassanii*) is an obligate cave‐dwelling bat with a restricted distribution in south‐eastern Australia. The national recovery plan for the southern bent‐winged bat recommends investigating and developing techniques that would enable PIT technology to be used for quantifying age‐ and sex‐structured survival rates and to help identify the cause of population decline (Lumsden & Jemison, 2015), whilst minimizing trapping occasions and disturbance. Bent‐winged bats in south‐eastern Australia generally favor large caves with relatively large entrances (Dwyer, 1963) and do not readily accept cave gates (Slade & Law, 2008; Thomson, 2002). Therefore, modifying cave entrances to detect PIT‐tagged southern bent‐winged bats were deemed to pose an unacceptable risk to the species. As a result, there was a need to develop a system that could detect bats as they flew through large passages, without impacting their behavior.

Here we describe the challenges and successes of using large PIT antenna configurations for monitoring a small, volant, and fast‐moving organism, the southern bent‐winged bat, over a three‐year period. We PIT‐tagged and monitored 2,966 individuals, optimized an RFID system to successfully meet our aims of high detectability and low impact, and provide recommendations for other researchers considering using this technology for other wildlife species.

## 2 METHODS

### 2.1 Study sites

Our study was based at two limestone caves used by the southern bent‐winged bat in south‐east South Australia. From spring to autumn, southern bent‐winged bats form a large colony at their primary maternity cave, Bat Cave, in the Naracoorte Caves National Park, World Heritage Area. This was our primary study site. It is a horizontal cave system with a roof window entrance measuring approximately 7 m by 4 m. Mains (240 V AC) electricity is connected to the cave to power permanent infrared and thermal cameras located inside the cave. These cameras transmit live images of the bats to the nearby Bat Observation Centre for visitor tours which form part of the tourist attractions for the national park. The secondary study site was a nonbreeding cave located on private property near Glencoe, South Australia, about 72 km from Bat Cave. The entrance of this cave measures approximately 6 m wide by 2 m high and is fenced from livestock. The southern bent‐winged bat is the only bat species that is known to roost in these caves.

### 2.2 RFID systems and installation

The radio‐frequency identification (RFID) system used in this study was the Biomark IS1001—a low frequency (134.2 kHz) system with dynamic, automatic tuning. The system consists of a reader, data logger, and 15‐m flexible cord antenna that collectively powers, detects, and records PIT‐tags. The antenna is intended to be configured as a loop that detects tagged individuals as they pass through the loop. All data are recorded as log files to the internal memory or a USB flash drive connected to the data logger. We coupled this system with Biomark high performance 12.5 mm FDX‐B tags (HPT 12) which are reported by the manufacturer to provide a greater read‐range than other PIT‐tags of the same size.

The entrance of Bat Cave was too large for the 15 m antenna. The narrowest section of the cave (hereafter, referred to as “the restriction”) is located approximately 100 m from the entrance, measures approximately 5 m wide and up to 2.8 m high, and was identified as the most suitable position for the RFID system. Despite the distance from the cave entrance, we assumed that bats would fly through the restriction when present at Bat Cave because the restriction is a high traffic area for bat movement. Individuals fly through this passage to access the three main roosting chambers, including the maternity chamber where the bats raise their young (Dwyer & Hamilton‐Smith, 1965). Individuals also move through the restriction during the day to drink from dripping stalactites in the “drinking chamber”, located in an alcove off the main passage between the entrance and the maternity chamber (Codd, Clark, & Sanderson, 1999).

**Figure 1:**
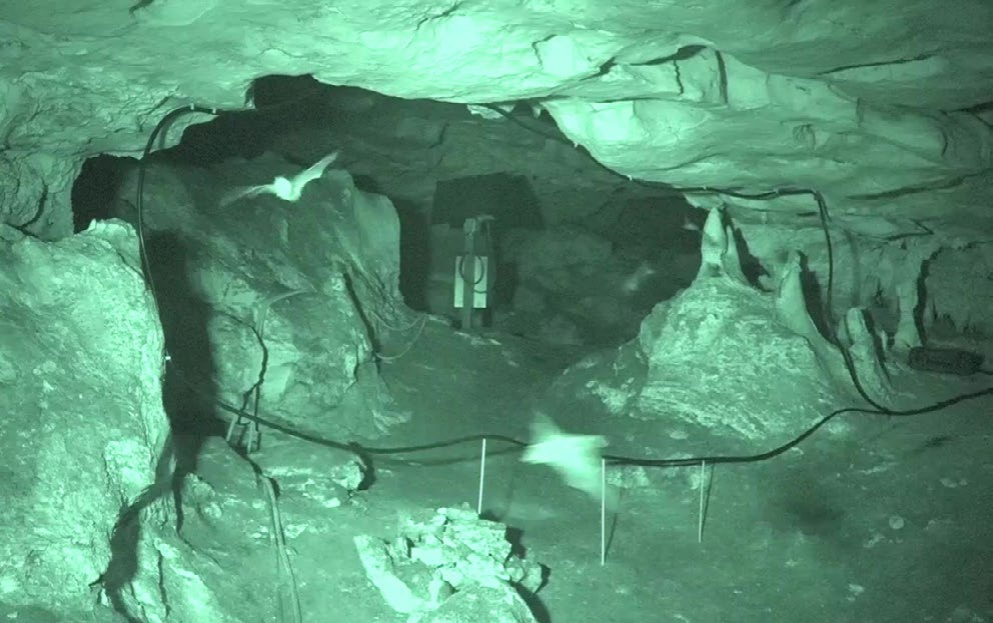
Southern bent‐winged bats flying through the 15 m loop antenna which was installed at the restriction in Bat Cave. The bottom of the antenna loop was raised above the cave floor. Only a small area of the restriction (to the right of the stalagmites) was not included in the detection space. Note that the “tail” of excess antenna cord was laid together and leads to the RFID reader on the right‐hand side of the image. Boxes containing the batteries, charger, and battery‐switching unit are located to the right of the camera's field‐of‐view. The structure in the middle of the photograph is a decommissioned infrared camera which provided real‐time footage to the Bat Observation Centre prior to our study—other cameras are still in operation in other parts of the cave

The placement of the antenna at the restriction in Bat Cave needed to satisfy two conditions: firstly, that bats would not collide with the antenna nor have their flight paths altered, and secondly, that the antenna configuration gave the best coverage and sensitivity for reading the tags. Despite the length and flexibility of the antenna, the Biomark IS1001 cord system will not successfully create a detection field in all configurations. Large rectangular antenna configurations (with a width exceeding 2.5 m) are likely to obtain the greatest antenna sensitivity by minimizing the height of the rectangle as much as possible (ideally to 1 m) and by laying the excess antenna cable close together (K. Pomorin, Karl Tek, pers. comm.). As such, the dimensions of the restriction at Bat Cave were substantially larger than those advised for successful PIT‐tag detection. To determine the optimal antenna placement and configuration, we first observed the flight path of the bats through the restriction for several hours (including during a dusk fly‐out) in August 2015, using a thermal camera (FLIR Photon 320). Analysis of the footage demonstrated that the bats flew in the upper‐half of the restriction. It was therefore determined that the cord antenna could be safely configured with the bottom of the antenna setup to 1 m above the cave floor, thereby creating a more desirable height for the rectangular antenna configuration. As metal can interfere with antenna performance (Biomark Inc, 2015; Freeland & Fry, 1995), the cord antenna was attached to the wall and ceiling of the cave using plastic saddle clips drilled into the limestone. The bottom of the antenna was supported off the ground with flexible fiberglass poles which were drilled into the cave floor and fastened using plastic cable ties. The final dimensions of the antenna were a maximum of 4.8 m wide and 1.8 m high (Figure 1). The excess cord of the antenna was laid together in parallel (touching, or close to touching) and kept in place with cable ties.

The system was connected in January 2016 and powered using two battery banks because the Biomark IS1001 RFID system is not compatible with Australian 240 V AC mains power. Each battery bank was comprised of two deep‐cycle 12 V DC batteries run in series to create an output of 24 V DC. The batteries were charged by a battery charger (CTEK MXT 14) connected to the mains power supply in the cave. The charger and batteries were separated from the RFID system by a battery‐switcher unit (Biomark standard battery‐switcher) that switches between charging and drawing power from each of the battery banks on a three‐hour rotation. This system is designed to sustain the life of the batteries by ensuring the batteries are not drawn too low and to keep the RFID system electrically isolated from mains power so that it did not interfere with the system's performance. The batteries, charger, and battery‐switcher were placed in plastic tubs with ventilation holes.

After the antenna was installed, the restriction was monitored with a video camera (Sony HDR‐CX900E used with an infrared illuminator) over three nights in January 2016. No avoidance behavior was observed, with bats flying through the loop unhindered. No bats were seen evading detection by going under or to the side of the antenna.

**Figure 2:**
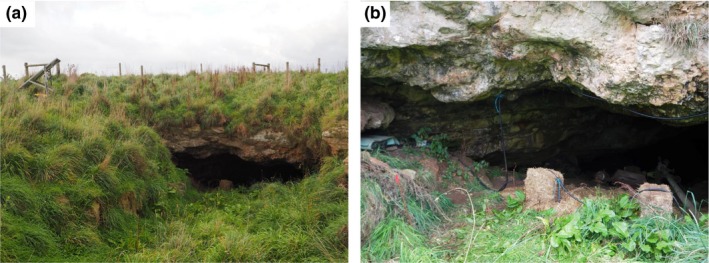
The Glencoe study site, with the RFID system and associated solar setup installed at the cave entrance. (a) Overall layout and shape of the entrance, and proximity of the solar installation (on the left of the image). (b) Close up of the left side of the cave entrance, showing: the Biomark IS1001 (covered with a foam matt for added protection) under a rock ledge on the left of the image; the flexible cord antenna in place around the mouth of the cave; the straw‐bales used to raise the antenna; disused metal irrigation infrastructure just within the lip of the cave; and the red tip of the copper earthing rod in the bottom left corner

A second RFID system was installed at the cave near Glencoe in April 2017 (Figure 2). The antenna was installed at the mouth of the cave using plastic saddle clips drilled into the limestone and raised off the ground using small rectangular straw‐bales and plastic cable ties. The RFID system was powered by a single battery bank and comprised of two AGM 100 AH deep‐cycle 12 V batteries (RITAR RA12‐100) run in series. The batteries were charged by a 265‐watt polycrystalline solar panel (Hanover HS265‐30) and a 12/24 V 20 A solar charge controller (Projecta SC320). The solar panel and controller were installed 6 m from the cave entrance. To protect it from weather, the battery bank was placed within a heavy‐duty plastic tub underneath the tilted solar panel. The 6 m of cabling between the RFID system and the solar controller was protected with PVC conduit. The final dimensions of the antenna were a maximum of 4.5 m wide by 1.7 m high.

### 2.3 Trapping and tagging

We trapped and PIT‐tagged bats at Bat Cave in January and February over three consecutive years, 2016–2018. Bats were trapped with Austbat harp traps (Faunatech, Mount Taylor) set exterior to the fence that surrounds the cave entrance. Each bat was PIT‐tagged using a sterilized 12‐gauge needle (Biomark MK10 implanter and N125 needles in 2016, and Biomark MK 25 Implant Guns and HPT12 Pre‐load Trays in 2017 and 2018). The 12‐mm tag (Biomark HPT 12) was injected subcutaneously so that the tag rested between the scapulae, and the injection site was sealed with a drop of surgical glue (3M™ VetBond™) to minimize tag loss (Lebl & Ruf, 2010). A total of 2,966 southern bent‐winged bats were tagged over the course of the study (approximately 1,000 per year). During the handling and tagging, the bats typically remained calm and were released minutes after the procedure (van Harten et al., 2019). All trapping, handling, tagging, and data collection procedures were approved by the La Trobe Animal Ethics Committee (AEC15‐67) and the South Australian Department of Environment and Water (U26453).

### 2.4 Data collection

The Biomark IS1001 data logger recorded two types of data chronologically into daily log files: tag data and system data. Each tag detection is recorded with the exact time and date of detection and the PIT‐tag's unique identification number. The system data records status and noise reports which include system settings and noise levels (i.e., unwanted signal). As a range of system settings can be chosen, the settings used in this study are provided in Appendix 1. Full status reports were generated by the system hourly and noise reports were recorded every five minutes. Data files were recorded directly to USB flash drives plugged into the data logger board. Data were collected from the study sites regularly (approximately monthly) by manually retrieving the flash drives. Other system maintenance included initiating a full tune of the antenna using the BioTerm program (Biomark) on a laptop connected to the RFID system via the mini USB port (undertaken approximately every two months) and the installation of a software update to both of the Biomark IS1001 units (undertaken once).

### 2.5 Quantifying and minimizing noise

Noise is the summation of unwanted in‐band frequency signals being received by the RFID system, including electromagnetic interference and natural environmental factors, which degrade system performance by competing with the tag signal. The Biomark IS1001 measures noise as “FDX‐B signal” in millivolts (0–900 mV range) and then converts this measurement into a percentage for ease of reference. At Bat Cave, initial noise levels were high (>25%). Potential sources for electromagnetic interference included the five pre‐existing thermal and infrared cameras situated in various chambers of the cave that were linked to the Bat Observation Centre. Associated with the cameras was a network of 240 V AC cabling. To find and eliminate the source/s of the noise, we turned off power to the cave, measured noise levels (by initiating a noise report with the BioTerm program), and then systematically turned back on each of the cameras and cabling networks. After each change, the read‐range and noise levels were recorded.

To decrease noise levels and increase system performance, we electronically earthed the RFID system at Bat Cave on 4 May 2016. The floor of the cave near the antenna is bed rock, with little to no available earth or soil. Dry limestone is a poor electrical conductor, so instead of drilling and inserting an earth rod into the floor of the cave, we buried a two‐meter copper earth rod horizontally under a thin bed of bat guano. The earth was connected to the exposed negative post‐terminal of the Biomark IS1001 with a saddle, 6 mm earthing cable and ring terminal. Two additional rods were attached in series with additional saddles and earthing cable in February 2017 in an attempt to strengthen the earth. An earth was also added to the system at Glencoe on 7 May 2018, by hammering a 1 m long earthing rod vertically into the soil near the entrance of the cave and then connecting the rod to the negative terminal of the Biomark IS1001.

### 2.6 Read‐range and the impact of noise

A standard measure of RFID system performance is read‐range, which we defined as the maximum horizontal distance from the loop antenna's vertical plane that a tag was detected. The greater the read‐range, the greater the total detection field and the less influence that angle and speed of the passing PIT‐tag has on the probability of a successful detection. Maximum read‐range was assessed by holding a test PIT‐tag and slowly moving it through the antenna loop at various points of the configuration. The read‐range was measured with a nonmetal measuring tape from the vertical plane of the antenna loop to the maximum perpendicular point that the tag was detected. Read‐range was measured after installation and after changes to the system setup or external conditions (e.g., potential noise sources).

As read‐range could only be measured in person at the study sites, we had limited capacity to measure the response of read‐range to the full variation in noise levels affecting the two systems. However, the Biomark IS1001 detects and records tag data at a rate of 30 “pings” per second, and so each flight of a tagged bat through the antenna loop is typically logged numerous times. We reasoned that, on average, larger read‐ranges (and hence larger detections fields) would result in more logged detections per detection event. To test our hypothesis that a negative relationship existed between noise and read‐range, we calculated the number of consecutive detections recorded for each bat pass and modeled this response variable as a linear function of noise using a zero‐truncated poisson regression, implemented with package VGAM with the R software for statistical computing, version 3.5.1 (R Core Team, 2018). All incidents of >50 consecutively logged detections of the same tag number were removed from this analysis, because occasionally very large numbers of consecutive detections were logged (e.g., thousands of detections) likely due to tagged bats roosting near the antenna.

### 2.7 Detection and encounter probability

We investigated detection probability in situ at Bat Cave with free‐flying bats tagged in early February 2017. After tagging, 209 bats were released at night in small batches within the cave, beyond the antenna, between the restriction and the maternity chamber. Each bat therefore needed to fly through the antenna at least once to exit the cave. Under the assumption that all 209 bats exited the cave after release, detection probability was estimated as the proportion of released individuals detected on the RFID system by midnight following their release, that is, bats were released after midnight, in the early hours of the morning and needed to be detected by midnight on the same date to be included in the proportion detected. This estimate is conditional on noise levels at the time of the experiment and can only serve as a coarse estimate of detection probability (the true detection probability for each animal pass is impossible to quantify directly).

We also used detection histories for each individual to consider how the daily probability of encounter varied with system noise and time of year, using data from the Bat Cave antenna system. To achieve this, we first derived capture–resight histories for each of the 2,966 PIT‐tagged bats, to produce a binary response variable (undetected/detected) for each individual across each day of the study period, with a “day” being defined as the 24 hr between successive middays. Using this variable, we identified the first and last detection event for each individual and derived a second binary variable indicating whether each individual was known to be alive. We calculated the daily encounter rate as the proportion of individuals known to be alive that were detected each day. We also used a binomial generalized additive model (R package “mgcv”) to estimate the per‐individual daily probability of encounter (i.e., the probability of being present at Bat Cave and being detected) as a function of noise (averaged for each day) and day of year. For the latter effect, we fitted a cyclic cubic regression spline to ensure continuity of the modeled response between the first and last day of the year.

## 3 RESULTS

### 3.1 System optimization and noise minimization

**Figure 3:**
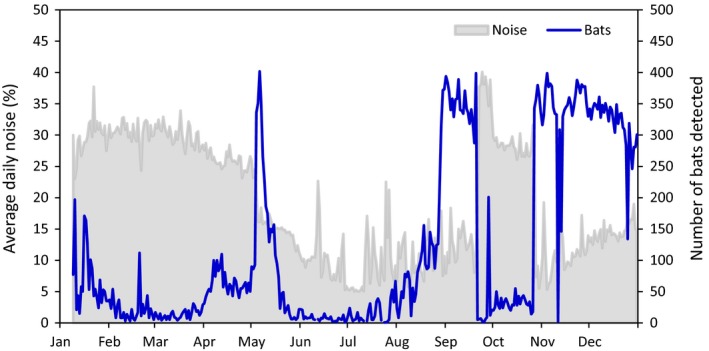
Average daily noise levels (%) and number of individuals detected (out of a possible 962 bats tagged) at Bat Cave in 2016. When an earth was installed on the RFID system at the beginning of May, there was a significant increase in the number of bats detected due to lower noise levels. Bats naturally dispersed from Bat Cave soon after earthing and began returning in August. High noise was inadvertently introduced when changes were made to a nearby thermal camera in late September. This dramatically decreased system performance and the number of bats detected. Minor improvements were made when the issue was discovered a week later, including tuning the antenna. The source of the interference was discovered after extensive trouble shooting in late October. The high noise ceased when the camera was unplugged, and the number of bats detected immediately returned to prior levels. A high noise event of unknown origin also occurred on a single date in mid‐November

Noise levels were a major factor in the RFID system performance. Earthing the system at Bat Cave decreased noise levels, increased read‐range (see section 3.2), and resulted in an immediate increase in the number of bats detected per day (Figure 3). Attaching a further two rods in series, on a later date, did not decrease noise levels further. A major source of noise (daily noise ~30%–40%) was inadvertently introduced in September 2016, when park management made changes to a thermal camera in the maternity chamber, approximately 50 m away from the RFID system. The interference caused major disruption to system performance with few bats being detected (Figure 3). The issue was resolved by disconnecting the camera; thereafter, average daily noise typically ranged between 5% and 18%.

**Figure 4:**
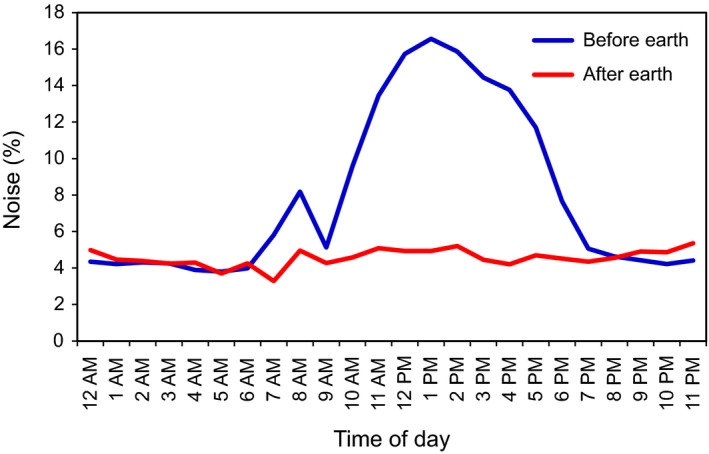
Mean hourly noise levels (%) at the cave at Glencoe before and after earthing, using all available data (2017–2018)

Noise levels recorded by the system installed at Glencoe were lower and less variable than at Bat Cave. The RFID system at Glencoe was initially powered directly from batteries and noise levels averaged 4%. After the solar panel and controller were installed to power the system for long‐term use, noise levels became more variable. There was a daily cycle, whereby average noise only exceeded 5% between dawn and dusk, with a peak at 1 p.m. (Figure 4). The likely source of this noise was the solar controller (which charged the batteries during daylight hours), but this was unlikely to have affected detection success since bat activity at Glencoe is typically recorded between dusk and dawn. Nevertheless, as a precaution, the system was earthed in May 2018 which stabilized the noise levels throughout the day (Figure 4).

### 3.2 Read‐range and the impact of noise

**Figure 5:**
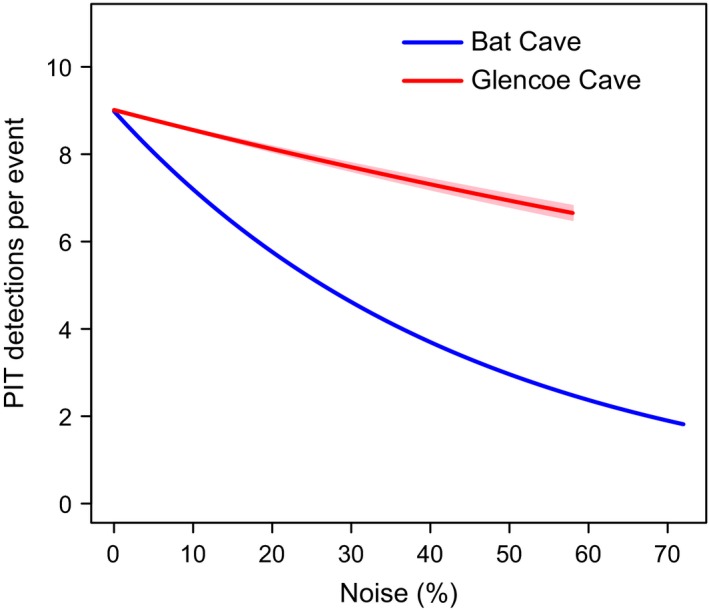
The negative relationship between average daily noise levels and the number of consecutive PIT detections per detection event at both study sites. The error margins are indicated in pink for Glencoe and are not visible for Bat Cave due to their very small size

The maximum read‐ranges measured at Bat Cave varied under different conditions over the study period and were negatively related to noise (Figure 5). The highest read‐range for this site (89 cm) was measured under testing conditions, when all mains circuits in Bat Cave were turned off, equating to a total detection field of more than 15 cubic meters. During the initial installation, maximum read‐range was just 30 cm near the antenna cable and the read‐range decreased toward the center of the antenna loop where there were large detection dead spots. Temporarily unplugging the battery charger from mains power for testing purposes increased maximum read‐range by an additional 20 cm. We therefore unplugged the battery charger during trapping trips in 2016 to detect as many bats as possible after their initial tagging. As a result, small peaks in the number of individuals detected during these trapping trips can be seen in Figure 3 (two in January, and one in late February). This was not required in subsequent years when performance issues had been resolved. After earthing the RFID system, read‐range increased to 58 cm across the antenna configuration with no dead spots. This further increased to 75 cm later in the study (from mid‐2017) when the cabling and protective conduit between the cameras in the maternity chamber and Bat Observation Centre were replaced and upgraded by cave management. At Glencoe, maximum read‐range was measured at 89 cm and increased to 105 cm after earthing, with no dead spots.

### 3.3 Detection and encounter probability

The number of individuals tagged in the study and subsequently detected on the system at Bat Cave was lowest in the first year at 92.3%, when noise levels were higher, and improved in the following years when noise levels were lower. In 2017 and 2018 these rates were 99.1% and 98.7%, respectively, of the bats tagged in that year—with 95.7% and 94.6% still detected >10 days after tagging. In total, 2,875 of the 2,966 (96.9%) tagged individuals were detected at least once.

The trial in 2017 where tagged bats were released within Bat Cave past the RFID system revealed that not all bats that were assumed to have flown through the antenna were detected. A total of 154 of the 209 bats were detected by midnight on the date of their release. This indicated an apparent probability of a tagged bat being detected at least once whilst present at Bat Cave (on a given date with average daily noise at 15%) at 0.74. Of the bats not detected (*n* = 55), 94.5% were detected on a later date (hence removing the possibility of death and tag loss for nondetection of these individuals), and just three individuals were never detected at Bat Cave or Glencoe.

**Figure 6:**
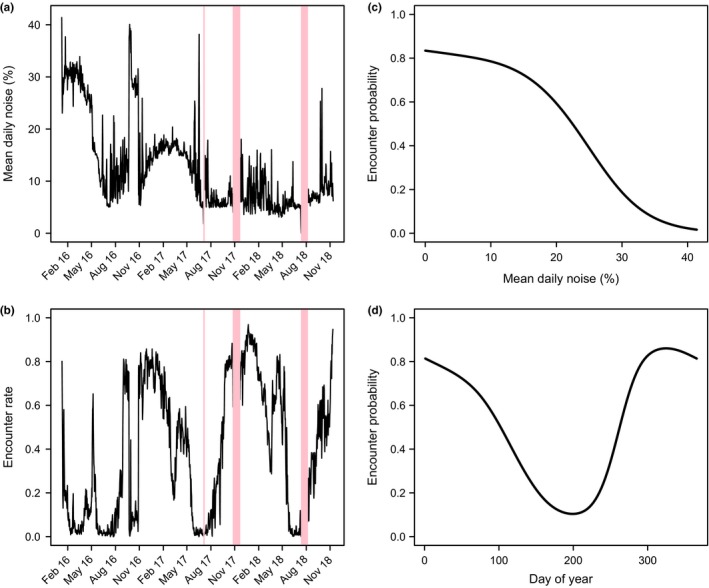
Encounter probability models of tagged individuals at Bat Cave in relation to noise levels and day of year. (a) and (b) show the fluctuating noise levels and encounter rates (i.e., proportion of bats detected that are known to be alive) by day of year, with pink bars indicating power outages when no data were recorded on the RFID system. (c) Encounter probability in relation to noise levels (when day of year = 1); (d) Encounter probability throughout the year (using noise levels at 5%). Earthing occurred in early May 2016

At Bat Cave, the daily encounter probability per individual (i.e., the probability that a tagged bat was both present and detected) reflected both the effect of varying noise levels and seasonality of bat congregation at the maternity cave (Figure 6). Encounter rate and probability peaked at >0.8 over the breeding season when larger numbers of bats congregated at the maternity site and decreased over winter coinciding with natural dispersal to nonbreeding roost sites. Infrared video monitoring (using the existing cameras in Bat Cave) confirmed that the decline in bat numbers over winter was real, and not due to detection problems, with few bats being observed until August, when clusters of thousands of bats began reforming at the maternity cave. Higher noise levels decreased encounter probability overall; however, there was particularly pronounced drop in encounter probability once daily average noise levels exceeded 15% (Figure 6c), for example, model‐based estimates (when day of year = 1) were 0.59 (95% CI [0.590, 0.597]), 0.39 (95% CI [0.385, 0.393]), and 0.19 (95% CI [0.184, 0.0191]) when noise was 20%, 25% and 30% respectively, compared to 0.72 (95% CI [0.721, 0.726]) at 15%. Encounter probability on the date of the release experiment outlined above (when day of year = 35 and noise levels were 15%) was 0.66 (95% CI [0.655, 0.660]).

**Figure 7:**
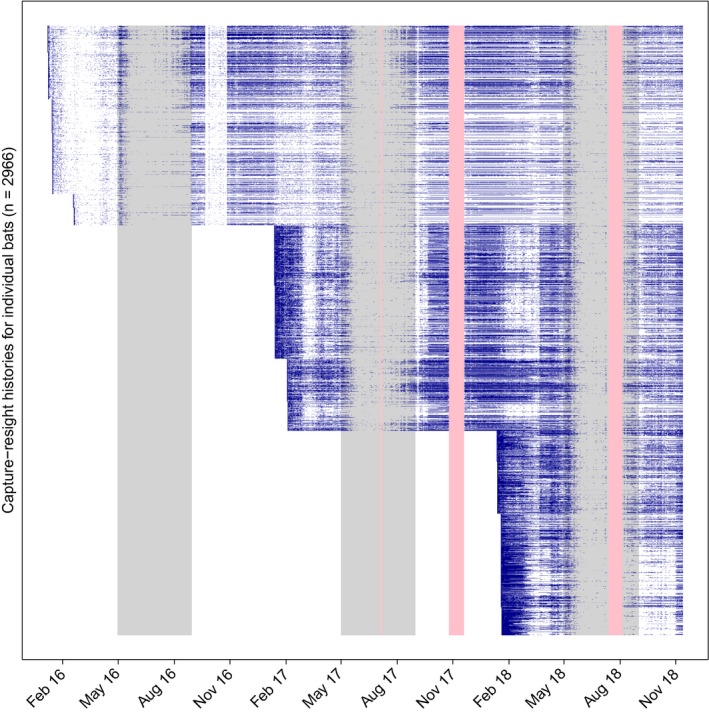
Capture–resight histories of all PIT‐tagged individuals at Bat Cave over the three‐year study. Each of the 2,966 tagged bats is represented as an individual row on the *y*‐axis, with initial capture and subsequent daily detections/presence at Bat Cave marked in blue. The data occur in blocks because individuals were tagged over 3 years and seven trapping events. Absence (white) could be due to death, tag loss, migration to other cave locations, or lack of detection (especially due to noise problems such as those encountered before earthing in early May 2016 and during the high noise event in October–November 2016). The pink shading indicates missing data due to power outages and gray shading indicates May to August, when bats typically disperse away from Bat Cave

High encounter rates and high RFID system sensitivity meant that data accumulated quickly. More than 17.8 million PIT‐tag detections were logged over the course of the study. Collapsing consecutive tag detections of the same tag number (representing a single pass through the antenna) resulted in 1,304,784 unique detection events at Bat Cave and 129,284 at Glencoe. The number of unique individuals detected per day at Bat Cave ranged between 0 and 1743, and up to 534 for Glencoe. Tagged individuals were detected over multiple seasons and years, with a high rate of return to Bat Cave after seasonal dispersal periods (Figure 7).

## 4 DISCUSSION

A limitation of PIT‐tag technology for wildlife research has been the short read‐range capabilities of PIT antennas (Gibbons & Andrews, 2004). With the installation of large RFID antenna systems at southern bent‐winged bat roosting caves, we have demonstrated that antenna dimensions and read‐range distances can reach greater magnitudes than previously described. Earlier studies using PIT technology at bat roosts or water sources described read‐ranges as small as 5–15 cm (Adams & Hayes, 2008; Neubaum et al., 2005), including with the same antenna as used in our study but in a different configuration and setup (Adams & Ammerman, 2015). The greatest read‐range we found in the literature for a PIT antenna was 35 cm using a plate antenna (Norquay & Willis, 2014). We have shown that large loop style antenna configurations can achieve read‐ranges up to 105 cm on both sides of the detection plane. These results demonstrate greater flexibility of applications for PIT technology to study a wider range of organisms, many of which could not be studied with this technology previously, including many cave‐dwelling bat species.

We had high overall detection success, particularly in the second and third years when performance of our RFID system was optimized. Across the full study period, 97% percent of bats were detected at least once. This compares with 76% (Adams, 2015), 67% (Adams & Hayes, 2008) and 62% (Horn, 1998) of bats PIT‐tagged in shorter‐term bat studies, and 65% of tagged juveniles and 77% of adult females successfully detected in a longer‐term study over four years and multiple roost sites (Ellison, O'Shea, Neubaum, Neubaum, et al., 2007). Factors that may have contributed to the higher overall detection success in our study likely include the advancement in technology used, concerted efforts made to monitor and increase RFID system performance, and the behavior of southern bent‐winged bats that show high fidelity to the Bat Cave site and reliably congregate at this maternity cave in large numbers.

Compared with traditional microbat marking and trapping methods, the use of small PIT antennas at roost sites has been demonstrated to significantly increase “recapture” probability and the accuracy of survival estimates, without incurring the cost of increased disturbance from re‐trapping (Ellison, O'Shea, Neubaum, Neubaum, et al., 2007). Our data obtained with large antennas likewise demonstrate high rates of passive detection success over time. The magnitude of the data demonstrates exciting possibilities for future research, which could answer important ecological questions to inform threatened species recovery, such as survival rates, as well as behavior, movement, and migration patterns. Whilst our testing with hand‐released bats demonstrated imperfect daily detection rates, mark–recapture methods assume detection/recapture probabilities <1; consequently, this is not usually a problem unless recapture rates are very low (Waller & Svensson, 2016).

A key finding from this study is that large PIT antennas are highly sensitive to noise (unwanted signal) levels. Bat Cave system was notably affected by noise introduced by the power supply. Before earthing the RFID system, unplugging the system's battery charger from mains power increased read‐range by an additional 20 cm. This was despite the RFID system running on batteries and the battery‐switcher unit separating the RFID system from the battery charger and associated mains power. Earthing mitigated this issue; however, total collapse in detection capacity resulting from a thermal camera installed 50 m from the RFID system demonstrates the sensitivity of the system to unexpected noise sources, even after earthing. Noise levels are under constant flux and can be affected by a wide range of man‐made and natural environmental factors (e.g., atmospheric noise; ITU, 2016), as such, not all noise sources could be identified or removed and over the course of the study, noise levels fluctuated over minutes, hours, days, and seasons.

Our encounter probability model demonstrated that noise levels and seasonal activity patterns of the bats (time of year) were major factors influencing detection (Figure 6). Encounter probability (the likelihood that a bat was both present at Bat Cave and successfully detected) was just 0.08 lower than the proportion of bats detected in the release experiment (under known presence). The relatively narrow difference between these two measures may have been due to the timing of the release experiment, which occurred at peak season at Bat Cave, when most bats are thought to be present at the maternity cave (Dwyer & Hamilton‐Smith, 1965). Whilst the release experiment was undertaken only on one occasion, the similar results during high levels of presence at Bat Cave suggest that noise level and time of year may be suitable proxies in our model for other factors affecting detection of our tagged population, such as behavior of the bats, and speed and angle of the tags as they pass through the antenna.

Early in the project, we conducted preliminary testing to attempt to quantify and predict the effect of speed and angle on detection success; however, this proved difficult and was inconclusive. A major issue was that factors affecting detection were interdependent; for example, slightly different antenna configurations, environment, or noise levels altered the level to which speed and angle affected detection. Furthermore, replicating natural bat flight was difficult, and recording equipment (such as cameras) used to record the experiments introduced electromagnetic interference, which altered detection outcomes. Using noise metrics as a covariate in analysis may be a way around these issues, because noise is a major determinant of read‐range (Figure 5). Given that PIT readers detect tags at a fixed rate per second, the larger the detection field, the faster a tag should be able to pass through the antenna and still be detected. Additionally, greatest read‐ranges are achieved with the RFID system when tags pass perpendicular to the detection plane (K. Pomorin, pers. comm.). Ad hoc experimentation upon setting up the antenna confirmed that passing a test tag through the antenna at increasing angles from perpendicular to the antenna plane dramatically decreased the maximum read‐range (E. van Harten, pers. obs.). In fact, holding a test tag parallel to the antenna plane resulted in the tag not being detected at all. Therefore, lowered read‐range due to elevated noise would likely compound angle issues, whilst greater read‐ranges should allow for a greater range of angles for passing tags. In our study, read‐range is likely important for accommodating the natural flight behavior of southern bent‐winged bats and may explain the notable differences in encounter probability with small (e.g., 5%) increases in noise levels (Figure 6c). We have found little literature examining these or other factors affecting PIT‐tag detection success (but see Freeland & Fry, 1995 for close‐range detection, using hand‐held PIT‐tag scanners), and we therefore suggest that further investigation into this area is warranted.

Our secondary system at Glencoe was less prone to noise issues than Bat Cave and recorded higher read‐ranges throughout the study. However, even under low noise levels (e.g., 5%) at both sites, read‐range (and detections per event) was higher at Glencoe (Figure 5). The higher read‐ranges at this study site were therefore not due to average daily noise levels alone. Other factors that may have contributed to the greater read‐ranges at Glencoe may be the slightly smaller antenna configuration and that higher noise levels only occurred during the day, when bats were not passing through the antenna to enter or exit the cave (Figure 4). We were initially concerned that metal infrastructure located at the entrance to the cave at Glencoe, including old irrigation pipes and pumping equipment (Figure 2), might interfere with system performance and successful tag detection. The effect of metal disturbing RFID has been experimentally demonstrated using hand‐held PIT‐tag readers (Freeland & Fry, 1995) and is highlighted as a potential noise source in the Biomark IS1001 user manual (Biomark Inc, 2015). However, the presence of this metal did not appear to cause any notable issues in our study. Unlike Bat Cave, Glencoe was free from nearby electronic equipment and cabling within 100 m of the setup, except for electric fencing and our RFID and solar systems themselves. In most instances, RFID systems can be charged with solar power rather than requiring access to mains power. Our Glencoe study site may therefore provide a more typical example of the potential performance of large PIT antennas for other studies. Unfortunately, at Bat Cave we were unable to bypass mains power by using solar due to the RFID system needing to be placed within the cave, approximately 100 m from the entrance and sunlight. However, our results offer the opportunity to contrast the performance of large PIT antennas in two different environments and setups, which can assist other researchers make informed decisions to optimize their methods.

Based on our study, we make seven recommendations for the use of large PIT antennas:

1. Monitor animal behavior before and after installation to ensure that antennas do not cause adverse effects on wildlife (such as avoidance behavior or collisions);
2. Minimize the presence of electronic equipment, devices and cabling within 100 m of the antenna;
3. Where possible, choose necessary devices and power supply (such as solar controllers) that emit low noise levels;
4. Earth large antenna systems as a standard protocol to optimize system performance;
5. Experiment with antenna configuration (including the placement of excess antenna length) to achieve maximum read‐range, as small configuration changes can have significant effects;
6. Continually monitor noise levels, because noise levels fluctuate naturally, and can be influenced by unexpected sources; and
7. Quantified noise metrics should be included as covariates influencing the probability of detection in subsequent statistical analysis of marked populations.

Overall, compared to alternative methods, PIT‐tagging appears to be a safe marking method with favorable benefits to the study population, such as reduced disturbance by minimizing trapping events and low tag weight. Importantly, this technique boasts high re‐detection rates and therefore can yield large volumes of continuous data over multiple seasons and years. Whilst the initial cost of equipment may appear as a limitation, this is offset by the comparatively low cost of subsequent re‐detections of individuals over the course of a study, especially for larger studies such as ours. One limitation to current PIT‐tag studies is the maximum length and potential configurations of commercially available cord antennas. The results of our study, using a 15 m antenna, suggest that even longer antennas may be successfully configured to cover larger entrances. At the time of writing, we have had some preliminary success detecting tagged bats at Bat Cave entrance using a third (specially ordered) 22 m antenna, and as technology progresses options are likely to continue to diversify. Our study demonstrates that large PIT antennas can successfully be used for long‐term studies to monitor small, volant, fast‐flying animals that move across large distances. The availability of large antennas with larger detection fields increases the potential applications of this technology, and consequently, we believe that the full potential of PIT‐tag technology as an ecological research tool is yet to be realized.

## CONFLICTS OF INTEREST

The authors have no conflicts of interest to declare.

## AUTHORS CONTRIBUTIONS

E.v.H., R.L., L.F.L., N.M. and T.R. conceived and designed the study; E.v.H. led the trapping and tagging field work with assistance of L.F.L. and T.R.; E.v.H. and T.R. installed the RFID systems, troubleshooted system problems and downloaded data; E.v.H., T.A.A.P., T.R. and J.W. manipulated, explored and analyzed the data; all authors contributed to data interpretation; E.v.H. drafted the paper; and all co‐authors contributed to revising the manuscript.

## Data Availability

Data used in these analyses are available at Dryad https://doi.org/10.5061/dryad.vh8t993.

## APPENDIX 1

An example of a Full Status Report from the Biomark IS1001 installed at Bat Cave including the system settings chosen for this study.

**Table:**

INF: Start of full status report	
Reader	
ID	01
Model	IS1001
S/N	1,442.0909
Date	11/12/2018
Time	00:28:35
Application firmware version	1.6.2
Operation mode	Scan
Network mode	IS1001 Standalone
Exciter Sync. Mode	Sec. Master
Date/Time Sync.	Disabled
Beeper	Disabled
Tag display format	DEC
Initiation delay	Disabled
Reader auto standby voltages	16 V, 18 V
Idling time	Disabled
Alarms	
Antenna current low alarm	1.0 A
Noise high alarm	40%
Tuning capacit. high alarm	970
Tuning capacit. low alarm	50
Alarms unique delay	60 s
Antenna/tuning	
Exciter voltage level	5
Dynamic tuning	Enabled
Tuning target phase	393
Tuning target phase deviation threshold	9
Measurements	
Antenna current gain	120
Antenna current offset	110
Communication	
Local port speed	115,200
Tags to local port	Enabled
Alarms to local port	Enabled
Messages to local port	Enabled
Remote port protocol	ASCII
Detection	
HDX tag detection	Disabled
Fastag detection	Disabled
Detection counter enabled	Yes
Unique mode	Disabled
Unique delay	60 s
FDXB detection scan time	120 ms
VTT level	128
Auto VTT delay	60 min
Memory	
Tags memory size	100,015
Status reports memory size	1,023
Save tags to memory	Disabled
Save VTT To memory	Enabled
Save stat. reports to memory	Enabled
Reports	
Auto noise report delay	5 min
Auto status report delay	60 min
Diagnostics	
Detection counter	369,491
Tags in memory	36,529 (36%)
Status reports in memory	1,020 (99%)
Input voltage	24.0 V
Exciter voltage	19.8 V
Antenna tuning	Tuned
Antenna current	5.2 A
Tuning capacitors	76
Tuning phase	399
Tuning relative phase	−6
FDXB signal level	48 mV (5%)
Temperature	23.2 C
Sync. input present	No
Sec. master active	Yes
Active alarms	
SRP: 01 11/12/2018 00:28:35.100 16,0,1,0,5,‐6,76,36,99,240,198,52,5,399,232,21,0,0,0,0	
INF: End of full status report	

## References

[ece35482-bib-0001] Adams, E. R. (2015). Seasonal and nightly activity of Mexican long‐nosed bats (*Leptonycteris nivalis*) in Big Bend National Park, Texas. Masters thesis, Angelo State University.

[ece35482-bib-0002] Adams, E. R. , & Ammerman, L. K. (2015). A serpentine antenna configuration for passive integrated transponder tag readers used at bat roosts. The Southwestern Naturalist, 60, 393–397. 10.1894/0038-4909-60.4.393

[ece35482-bib-0003] Adams, R. A. , & Hayes, M. A. (2008). Water availability and successful lactation by bats as related to climate change in arid regions of western North America. Journal of Animal Ecology, 77, 1115–1121. 10.1111/j.1365-2656.2008.01447.x 18684132

[ece35482-bib-0004] Aldridge, H. D. J. N. , & Brigham, R. M. (1988). Load carrying and manoeuvrability in an insectivorous bat: A test of the 5% ‘rule’ of radio‐telemetry. Journal of Mammalogy, 69, 379–382. 10.2307/1381393

[ece35482-bib-0005] Allen, A. A. (1921). Banding bats. Journal of Mammalogy, 2, 53 10.2307/1373683

[ece35482-bib-0006] Baker, G. B. , Lumsden, L. F. , Dettmann, E. B. , Schedvin, N. K. , Schulz, M. , Watkins, D. , & Jansen, L. (2001). The effect of forearm bands on insectivorous bats (Microchiroptera) in Australia. Wildlife Research, 28, 229–237. 10.1071/WR99068

[ece35482-bib-0007] Bino, G. , Kingsford, R. T. , Grant, T. , Taylor, M. D. , & Vogelnest, L. (2018). Use of implanted acoustic tags to assess platypus movement behaviour across spatial and temporal scales. Scientific Reports, 8, 5117 10.1038/s41598-018-23461-9 29572497 PMC5865170

[ece35482-bib-0008] Biomark Inc. (2015). IS1001™ standalone operation user manual. Boise, ID: Biomarker Inc.

[ece35482-bib-0009] Blomberg, E. , Davis, S. , Mangelinckx, J. , & Sullivan, K. (2018). Detecting capture‐related mortality in radio‐marked birds following release. Avian Conservation and Ecology, 13, 5 10.5751/ACE-01147-130105

[ece35482-bib-0010] Bodey, T. W. , Cleasby, I. R. , Bell, F. , Parr, N. , Schultz, A. , Votier, S. C. , & Bearhop, S. (2018). A phylogenetically controlled meta‐analysis of biologging device effects on birds: Deleterious effects and a call for more standardized reporting of study data. Methods in Ecology and Evolution, 9, 946–955. 10.1111/2041-210X.12934

[ece35482-bib-0011] Britzke, E. R. , Gumbert, M. W. , & Hohmann, M. G. (2014). Behavioral response of bats to passive integrated transponder tag reader arrays placed at cave entrances. Journal of Fish and Wildlife Management, 5, 146–150. 10.3996/082012-JFWM-065

[ece35482-bib-0012] Castle, K. T. , Weller, T. J. , Cryan, P. M. , Hein, C. D. , & Schirmacher, M. R. (2015). Using sutures to attach miniature tracking tags to small bats for multimonth movement and behavioral studies. Ecology and Evolution, 5, 2980–2989. 10.1002/ece3.1584 26306181 PMC4542000

[ece35482-bib-0013] Codd, J. , Clark, B. , & Sanderson, K. (1999). Drinking by the common bent‐wing bat *Miniopterus schreibersii* and calcium in cave water. Bat Research News, 40, 9–10.

[ece35482-bib-0014] Cooke, S. J. , Nguyen, V. M. , Murchie, K. J. , Thiem, J. D. , Donaldson, M. R. , Hinch, S. G. , … Fisk, A. (2013). To tag or not to tag: Animal welfare, conservation, and stakeholder considerations in fish tracking studies that use electronic tags. Journal of International Wildlife Law & Policy, 16(4), 352–374. 10.1080/13880292.2013.805075

[ece35482-bib-0015] Dwyer, P. (1963). The breeding biology of *Miniopterus schreibersii blepotis* (Termminck) (Chiroptera) in north‐eastern NSW. Australian Journal of Zoology, 11, 219–240. 10.1071/ZO9630219

[ece35482-bib-0016] Dwyer, P. D. , & Hamilton‐Smith, E. (1965). Breeding caves and maternity colonies of the bent‐winged bat in south‐eastern Australia. Helictite, 4, 3–21.

[ece35482-bib-0017] Ellison, L. E. , O'Shea, T. J. , Neubaum, D. J. , & Bowen, R. A. (2007). Factors influencing movement probabilities of big brown bats (*Eptesicus fuscus*) in buildings. Ecological Applications, 17, 620–627. 10.1890/06-0315 17489265

[ece35482-bib-0018] Ellison, L. E. , O'Shea, T. J. , Neubaum, D. J. , Neubaum, M. A. , Pearce, R. D. , & Bowen, R. A. (2007). A comparison of conventional capture versus PIT reader techniques for estimating survival and capture probabilities of big brown bats (*Eptesicus fuscus*). Acta Chiropterologica, 9, 149–160. 10.3161/1733-5329(2007)9[149:ACOCCV]2.0.CO;2

[ece35482-bib-0019] Freeland, W. , & Fry, K. (1995). Suitability of passive integrated transponder tags for marking live animals for trade. Wildlife Research, 22, 767–773. 10.1071/WR9950767

[ece35482-bib-0020] Garroway, C. J. , & Broders, H. G. (2007). Nonrandom association patterns at northern long‐eared bat maternity roosts. Canadian Journal of Zoology, 85, 956–964. 10.1139/Z07-079

[ece35482-bib-0021] Gelling, M. , McLaren, G. W. , Mathews, F. , Mian, R. , & Macdonald, D. W. (2009). Impact of trapping and handling on leukocyte coping capacity in bank voles (*Clethrionomys glareolus*) and wood mice (*Apodemus sylvaticus*). Animal Welfare, 18, 1–7.

[ece35482-bib-0022] Gibbons, J. W. , & Andrews, K. M. (2004). PIT tagging: Simple technology at its best. BioScience, 54, 447–454. 10.1641/0006-3568(2004)054[0447:PTSTAI]2.0.CO;2

[ece35482-bib-0023] Godinho, L. N. , Lumsden, L. F. , Coulson, G. , & Griffiths, S. R. (2015). Network analysis reveals cryptic seasonal patterns of association in Gould's wattled bats (*Chalinolobus gouldii*) roosting in bat‐boxes. Behaviour, 152, 2079–2105. 10.1163/1568539X-00003315

[ece35482-bib-0024] Griesser, M. , Schneider, N. A. , Collis, M. , Overs, A. , Guppy, M. , Guppy, S. , … Hall, M. L. (2012). Causes of ring‐related leg injuries in birds – evidence and recommendations from four field studies. PLoS ONE, 7, e51891 10.1371/journal.pone.0051891 23300574 PMC3530577

[ece35482-bib-0025] Horn, J. W. (1998). Individual variation in the nightly time budgets of the little brown bat, *Myotis lucifugus*. Masters thesis, Boston University.

[ece35482-bib-0026] ITU (2016). Recommendation ITU‐R P.372‐13. Radio Noise. Geneva, Switzerland: International Telecommunication Union.

[ece35482-bib-0027] IUCN (2018). The IUCN red list of threatened species. Version 2018‐2. Retrieved from http://www.iucnredlist.org

[ece35482-bib-0028] Kerth, G. , & König, B. (1996). Transponder and an infrared‐videocamera as methods in a fieldstudy on the social behaviour of Bechstein's bats (*Myotis bechsteini*). Myotis, 34, 27–34.

[ece35482-bib-0029] Kerth, G. , & Reckardt, K. (2003). Information transfer about roosts in female Bechstein's bats: An experimental field study. Proceedings of the Royal Society of London. Series B: Biological Sciences, 270, 511–515. 10.1098/rspb.2002.2267 12641906 PMC1691266

[ece35482-bib-0030] Lebl, K. , & Ruf, T. (2010). An easy way to reduce PIT‐tag loss in rodents. Ecological Research, 25, 251–253. 10.1007/s11284-009-0629-y

[ece35482-bib-0031] Lemckert, F. , Brassil, T. , Kavanagh, R. , & Law, B. (2006). Trapping small mammals for research and management: How many die and why? Australian Mammalogy, 28, 201–207. 10.1071/AM06028

[ece35482-bib-0032] Lumsden, L. F. , & Jemison, M. L. (2015). Draft National Recovery Plan for the Southern Bent‐wing Bat *Miniopterus schreibersii bassanii*. Department of Environment, Land, Water and Planning, Victoria.

[ece35482-bib-0033] Lynn, S. E. , & Porter, A. J. (2008). Trapping initiates stress response in breeding and non‐breeding house sparrows *Passer domesticus*: Implications for using unmonitored traps in field studies. Journal of Avian Biology, 39, 87–94. 10.1111/j.0908-8857.2008.04204.x

[ece35482-bib-0034] Murray, D. L. , & Fuller, M. R. (2000). A critical review of the effects of marking on the biology of vertebrates In Boitani L., & Fuller T. K. (Eds.), Research techniques in animal ecology: Controversies and consequences (pp. 15–64). New York, NY: Columbia University Press.

[ece35482-bib-0035] Neubaum, D. J. , Neubaum, M. A. , Ellison, L. E. , & O'Shea, T. J. (2005). Survival and condition of big brown bats (*Eptesicus fuscus*) after radiotagging. Journal of Mammalogy, 86, 95–98. 10.1644/1545-1542(2005)086<0095:SACOBB>2.0.CO;2

[ece35482-bib-0036] Norquay, K. J. O. , & Willis, C. K. R. (2014). Hibernation phenology of *Myotis lucifugus* . Journal of Zoology, 294, 85–92. 10.1111/jzo.12155

[ece35482-bib-0037] O'Donnell, C. F. J. , Edmonds, H. , & Hoare, J. M. (2011). Survival of PIT‐tagged lesser short‐tailed bats (*Mystacina tuberculata*) through a pest control operation using the toxin pindone in bait stations. New Zealand Journal of Ecology, 35, 291–295.

[ece35482-bib-0038] O'Mara, M. T. , Wikelski, M. , & Dechmann, D. K. N. (2014). 50 years of bat tracking: Device attachment and future directions. Methods in Ecology and Evolution, 5, 311–319. 10.1111/2041-210X.12172

[ece35482-bib-0039] O'Shea, T. J. , Ellison, L. E. , Neubaum, D. J. , Neubaum, M. A. , Reynolds, C. A. , & Bowen, R. A. (2010). Recruitment in a Colorado population of big brown bats: Breeding probabilities, litter size, and first‐year survival. Journal of Mammalogy, 91, 418–428. 10.1644/08-MAMM-A-295.1

[ece35482-bib-0040] Perry, G. , Wallace, M. C. , Perry, D. , Curzer, H. , & Muhlberger, P. (2011). Toe clipping of amphibians and reptiles: Science, ethics, and the law. Journal of Herpetology, 45, 547–555. 10.1670/11-037.1

[ece35482-bib-0041] Pugh, M. , & Altringham, J. D. (2005). The effect of gates on cave entry by swarming bats. Acta Chiropterologica, 7, 293–299. 10.3161/1733-5329(2005)7[293:TEOGOC]2.0.CO;2

[ece35482-bib-0042] R Core Team (2018). R: computing. Vienna, Austria: R Foundation for Statistical Computing.

[ece35482-bib-0043] Reeder, D. M. , Kosteczko, N. S. , Kunz, T. H. , & Widmaier, E. P. (2004). Changes in baseline and stress‐induced glucocorticoid levels during the active period in free‐ranging male and female little brown myotis, *Myotis lucifugus* (Chiroptera: Vespertilionidae). General and Comparative Endocrinology, 136, 260–269. 10.1016/j.ygcen.2003.12.020 15028530

[ece35482-bib-0044] Rigby, E. L. , Aegerter, J. , Brash, M. , & Altringham, J. D. (2012). Impact of PIT tagging on recapture rates, body condition and reproductive success of wild Daubenton's bats (*Myotis daubentonii*). Veterinary Record, 170, 101 10.1136/vr.100075 22090155

[ece35482-bib-0045] Rosen, D. A. S. , Gerlinsky, C. G. , & Trites, A. W. (2018). Telemetry tags increase the costs of swimming in northern fur seals, *Callorhinus ursinus* . Marine Mammal Science, 34, 385–402. 10.1111/mms.12460

[ece35482-bib-0046] Safi, K. , König, B. , & Kerth, G. (2007). Sex differences in population genetics, home range size and habitat use of the parti‐colored bat (*Vespertilio murinus*, Linnaeus 1758) in Switzerland and their consequences for conservation. Biological Conservation, 137, 28–36. 10.1016/j.biocon.2007.01.011

[ece35482-bib-0047] Schlicht, E. , & Kempenaers, B. (2018). The immediate impact of ringing, blood sampling and PIT‐tag implanting on the behaviour of blue tits *Cyanistes caeruleus* . Ardea, 106, 39–98. 10.5253/arde.v106i1.a8

[ece35482-bib-0048] Schorr, R. A. , Ellison, L. E. , & Lukacs, P. M. (2014). Estimating sample size for landscape‐scale mark‐recapture studies of North American migratory tree bats. Acta Chiropterologica, 16, 231–239. 10.3161/150811014X683426

[ece35482-bib-0049] Slade, C. P. , & Law, B. S. (2008). An experimental test of gating derelict mines to conserve bat roost habitat in southeastern Australia. Acta Chiropterologica, 10, 367–376. 10.3161/150811008X414944

[ece35482-bib-0050] Soanes, K. , Vesk, P. A. , & van der Ree, R. (2015). Monitoring the use of road‐crossing structures by arboreal marsupials: Insights gained from motion‐triggered cameras and passive integrated transponder (PIT) tags. Wildlife Research, 42, 241–256. 10.1071/WR14067

[ece35482-bib-0051] Thomson, B. (2002). Australian handbook for the conservation of bats in mines and artificial cave‐bat habitats. Melbourne, Vic Australian Minerals & Energy Environment Foundation.

[ece35482-bib-0052] Toth, C. A. , Dennis, T. E. , Pattemore, D. E. , & Parsons, S. (2015). Females as mobile resources: Communal roosts promote the adoption of lek breeding in a temperate bat. Behavioral Ecology, 26, 1156–1163. 10.1093/beheco/arv070

[ece35482-bib-0053] Tuttle, M. D. (1979). Status, causes of decline, and management of endangered gray bats. The Journal of Wildlife Management, 43, 1–17. 10.2307/3800631

[ece35482-bib-0054] Unger, S. D. , Burgmeier, N. G. , & Williams, R. N. (2012). Genetic markers reveal high PIT tag retention rates in giant salamanders (*Cryptobranchus alleganiensis*). Amphibia‐Reptilia, 33, 313–317. 10.1163/156853812X641712

[ece35482-bib-0055] van Harten, E. , Reardon, T. , Holz, P. H. , Lawrence, R. , Prowse, T. A. A. , & Lumsden, L. F. (2019). Recovery of southern bent-winged bats *(Miniopterus orianae bassanii)* after PIT-tagging and the use of surgical adhesive. Australian Mammalogy. 10.1071/AM19024

[ece35482-bib-0056] Walker, K. A. , Trites, A. W. , Haulena, M. , & Weary, D. M. (2012). A review of the effects of different marking and tagging techniques on marine mammals. Wildlife Research, 39, 15–30. 10.1071/WR10177

[ece35482-bib-0057] Waller, J. , & Svensson, E. I. (2016). The measurement of selection when detection is imperfect: How good are naïve methods? Methods in Ecology and Evolution, 7, 538–548. 10.1111/2041-210X.12498

[ece35482-bib-0058] Weller, T. J. , Castle, K. T. , Liechti, F. , Hein, C. D. , Schirmacher, M. R. , & Cryan, P. M. (2016). First direct evidence of long‐distance seasonal movements and hibernation in a migratory bat. Scientific Reports, 6, 34585 10.1038/srep34585 27698492 PMC5048302

[ece35482-bib-0059] White, D. H. , & Seginak, J. T. (1987). Cave gate designs for use in protecting endangered bats. Wildlife Society Bulletin, 15, 445–449.

[ece35482-bib-0060] Wilmers, C. C. , Nickel, B. , Bryce, C. M. , Smith, J. A. , Wheat, R. E. , & Yovovich, V. (2015). The golden age of bio‐logging: How animal‐borne sensors are advancing the frontiers of ecology. Ecology, 96, 1741–1753. 10.1890/14-1401.1 26378296

